# Analysis and Comparison of Rad-Hard Ring and LC-Tank Controlled Oscillators in 65 nm for SpaceFibre Applications †

**DOI:** 10.3390/s20164612

**Published:** 2020-08-17

**Authors:** Danilo Monda, Gabriele Ciarpi, Sergio Saponara

**Affiliations:** Department of Information Engineering (DII), University of Pisa, 56126 Pisa, Italy; danilo.monda@phd.unipi.it (D.M.); gabriele.ciarpi@ing.unipi.it (G.C.)

**Keywords:** ring oscillator, LC-tank oscillator, SpaceFibre, rad-hard circuits, radiation effects, high-speed data transfer

## Abstract

This work presented a comparison between two Voltage Controlled Oscillators (VCOs) designed in 65 nm CMOS technology. The first architecture based on a Ring Oscillator (RO) was designed using three Current Mode Logic (CML) stages connected in a loop, while the second one was based on an LC-tank resonator. This analysis aimed to choose a VCO architecture able to be integrated into a rad-hard Phase Locked Loop. It had to meet the requirements of the SpaceFibre protocol, which supports frequencies up to 6.25 GHz, for space applications. The full custom schematic and layout designs are shown, and Single Event Effect simulations results, performed with a double exponential current pulses generator, are presented in detail for both VCOs. Although the RO-VCO performances in terms of technology scaling and high-integration density were attractive, the simulations on the process variations demonstrated its inability to generate the target frequency in harsh operating conditions. Instead, the LC-VCO highlighted a lower influence through Process-Voltage-Temperature simulations on the oscillation frequency. Both architectures were biased with a supply voltage of 1.2 V. The achieved results for the second architecture analyzed were attractive to address the requirements of the new SpaceFibre aerospace standard.

## 1. Introduction

Several thousand launch activities have been performed during the last half-century, and with the rapid development of technology, satellites are playing an important role in human society. These systems are widely used for navigation, communication, and earth observation. One of the first communication experiments with laser was conducted between two Low Earth Orbit (LEO) satellites and a geostationary satellite ARTEMIS. The experiment was performed with a data rate of up to 50 Mbps. Then, other experiments followed with an increased data rate to achieve inter-satellite communication links. Today, current trends in satellites show a rapid increase in data traffic and digital processing. The throughput of next-generation satellites for digital telecom applications, as well as scientific missions, surveillance, and remote sensing, will exceed terabits per second of data that must be processed on board. For instance, the high-resolution cameras and synthetic aperture radars need high-speed communications between the instruments and the on-board data storage system [1]. The optical technology, thanks to its high bandwidth-length product, the lightweight cabling, and electromagnetic hardness, can potentially be the solution for data-rate increment in satellites. In this direction, the European Space Agency (ESA) has recently released the new SpaceFibre standard for on-board satellite communication up to 6.25 Gbps [2,3]. The standard describes the very high-speed serial link and network technology, and it was designed specifically for use on-board spacecraft and satellites. This protocol provides a coherent quality of service mechanism able to support bandwidth reserved, scheduled, and priority-based qualities of service. SpaceFibre provides robust, long-distance communications for launcher applications and supports avionics applications with deterministic delivery constraints using virtual channels. Communication performances are strongly related to the ability to synchronize the receiver with the transmitter. This issue is typically fixed with a Clock Data Recovery (CDR), and the key block used for its synchronization is the Phase Locked Loop (PLL). The Voltage Controlled Oscillator (VCO) is the core system, inside the PLL, able to generate the required frequency of 6.25 GHz to be compliant with the SpaceFibre protocol. Although the required Total Ionizing Dose (TID) level is lower than 1 Mrad for space applications [4], the main problems are due to Single Event Effects (SEEs) that temporarily disturb the typical operation of the circuit. This work targets, as implementation technology, a commercial 65 nm CMOS from TSMC (Taiwan Semiconductor Manufacturing Company). This technology, thanks to its thin gate-oxide thickness, could be considered radiation hard up to few hundred Mrad TID levels, as proved in [5], and by us in previous designs of other high-speed circuits in [6,7,8]. To the best of the authors’ knowledge, in literature and market, there are not examples of rad-hard VCOs able to work at 6.25 GHz. The paper [9] showed the design of a PLL in the range from 0.2 GHz to 1.2 GHz, designed in 65 nm STMicroelectronics space technology. This system was irradiated up to 300 krad TID level, and its behavior was verified with different protons. In [10], a comparison between Ring Oscillator (RO) and LC-tank VCO for PLL was made for Large Hadron Collider’s (LHC) applications. Both were designed for a working frequency from 2.2 GHz to 3.2 GHz, and the SEE test performed with heavy-ions showed that the LC-VCO had a larger cross-section than the RO-VCO. Varactors have been identified as the most sensitive part of LC-tank architectures, and Triple Modular Redundancy (TMR) technique has been adopted to face SEEs in the design of the phase frequency divider. The goal of this work was to compare the performances of the widely used RO and LC controlled oscillators in radiation environments and to contribute with new approaches for exploiting the characteristics that have made these systems the most implemented.

This work is an extension of the preliminary work presented by us at the conference [11]. With respect to the conference presentation, this work presented the complete full custom design of schematic and layout for both the RO and the LC-tank controlled oscillator (respectively reported in Section 2 and Section 3). Moreover, this work in Section 4 provides transient and SEE simulations results, missing in [11]. Section 5 compares this work vs. the state-of-the-art. Conclusions are drawn in Section 6.

## 2. Ring Oscillator Based on a Cascade of Three Current Mode Logic (CML) Buffer

### 2.1. Ring Oscillator Schematic Design

**Figure 1:**
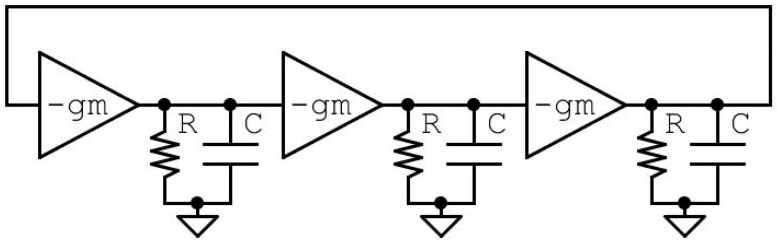
Ring oscillator modalized using inverting stage amplifiers.

The RO-VCO presented in this work is composed of a cascade of inverting amplifiers in closed-loop, as shown in Figure 1. The transconductance g_m_ is the gain of the single amplifier, while R and C are the equivalent output resistance and the equivalent input capacitance, respectively, of previous and following stages. According to Figure 1, the open-loop gain of the system composed of N generic stages is expressed as

(1) $$H\left(j\omega \right)\;=\;{\left(-\frac{g_{m}R}{1+j\omega RC}\right)}^{N}$$

For the Barkhausen oscillation criterion [12], the module of the transfer function has to be higher than one for the start-up condition and then equal to one to sustain the oscillation, while the transfer function phase has to be an integer multiple of 2π.

Applying this criterion at the model in Figure 1, we obtain the oscillation condition in terms of design parameters, expressed as

(2) $$g_{m}R\geq \frac{1}{cos\theta }$$

where *θ* is the phase shift introduced by each RC load, which for the Barkhausen oscillation criterion must be an integer multiple of π/N. In a ring oscillator, the frequency $f_{0}\;=\;1/2N\tau_{D}$, where $\tau_{D}$ is the delay of a single stage, and N is the number of stages in the loop. In order to limit power consumption and to reduce the silicon area to decrease the number of collisions caused by ionizing particles, *N* = 3 was chosen for the RO-VCO design. Although two stages ring oscillator provides a quadrature clock, as demonstrated in [13], a three stages oscillator is conventionally used for differential architecture [14]. Moreover, a smaller value of N provides a better phase noise [15] and a higher value of the working frequency $f_{0}$. With this choice, in accordance with Equation (2), the following condition is extracted as the main design guideline

(3) $$g_{m}R\geq 2$$

**Figure 2:**
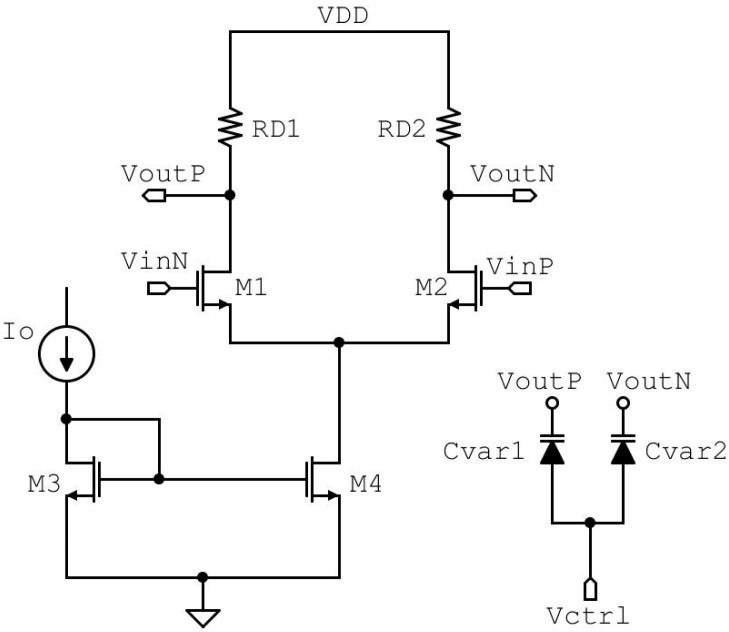
Circuit schematic of the single-stage, based on a Current Mode Logic (CML) buffer, of the ring oscillator and a couple of varactors connected at the two outputs.

Although CMOS architectures are largely used for their low static-power dissipation and high integration density, the designed RO-VCO is composed of three CML stages. The current mode logic architecture, based only on n-MOSFETs and resistors, is more suitable for high-frequency applications, thanks to their lower voltage swing and lower output impedance than a standard CMOS approach [16,17]. Moreover, the use of a differential structure allows obtaining higher common-mode disturb immunity than the use of a single-ended structure, as in classic CMOS circuits [18]. Guard rings and deep n-well are also used for the design of MOSFETs devices to prevent Single Event Latch-up (SEL) and to mitigate SEEs [19,20]. The single CML stage, shown in Figure 2, is made by a source coupled pair with a resistive load, a simple current mirror, and accumulation-mode MOSFETs varactors. Active components M1 and M2 are designed with the minimum channel length allowed by technology, and the transistor width is chosen in order to ensure, in the worst case, a g_m_*R value of 4, which is two times higher than the critical value expressed in Equation (3). The supply voltage for this technology is 1.2 V, and the value chosen for resistors shifts the output common-mode voltage level at 0.9 V. The RO-VCO bias current is controlled by the external generator I_0_ through the simple current mirror M3 and M4 with a unity current gain. These MOSFETs are designed with the maximum MOSFET length allowed by the RF-device model to increase the output resistance. A current of 4 mA feeds the controlled oscillator, and the post-layout simulated power consumption is 18 mW. In order to take control of the oscillation frequency, a couple of varactors are added at the output of each stage [21,22].

**Figure 3:**
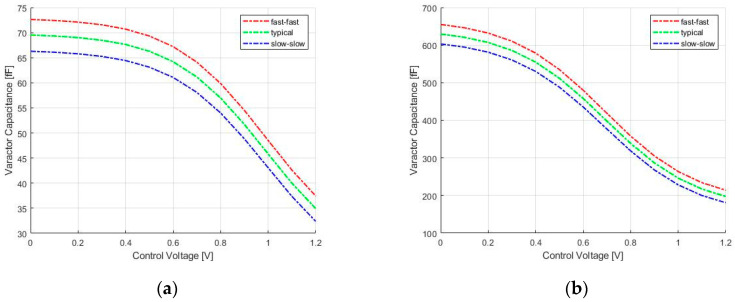
Varactor capacitance vs. control voltage for RO-VCO (**a**) and LC-VCO (**b**), different corner cases. RO, Ring Oscillator; VCO, Voltage Controlled Oscillator.

The frequency tuning is made, thanks to accumulation-mode MOSFETs devices. A single varactor is designed by 40 fingers divided into 2 groups, and each finger is designed with the minimum finger length of 200 nm and a finger width of 550 nm. They can assume the value in the range from 69.53 fF to 34.93 fF, respectively, for the minimum and maximum value of the control voltage in the typical case. As shown in Figure 3a, the variation of the capacitance value through the corner cases is lower than 5%.

The oscillation frequency of the RO-VCO based on a CML architecture is closely related to the value of the gate capacitance [23], and it is expressed by the relation $f_{0}\;=\;1/2\pi RC_{T}$, where R is the parallel between the pull-up CML resistive load and the output MOSFET resistance, while $C_{T}$ is the cumulative capacitance due by varactors and the gate capacitance of the following stage.

### 2.2. Ring Oscillator Layout Design

**Figure 4:**
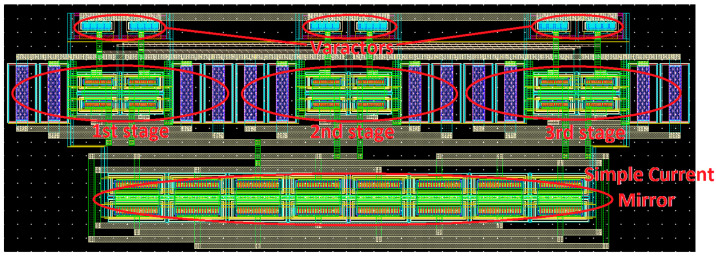
Full custom layout of the RO-VCO based on the CML buffer designed with Cadence Virtuoso [24].

The complete layout of the RO-VCO designed in 65 nm CMOS bulk-silicon technology is shown in Figure 4. The simple current mirror, in the bottom side, and the three source-coupled pairs are designed, adopting the common centroid technique to increase matching. All the gate terminals are turned in the same way so that the current flows in the same direction, and the space between instances is the minimum allowed by technology rules. A trade-off between metal width and length is made to prevent the electro-migration phenomena due to high current density. Moreover, alternate layers perpendicular to each other are drawn to minimize parasitic capacitances that lead to a frequency reduction. The total layout area of the proposed RO-VCO is 249 × 86 µm^2^.

## 3. LC-Tank Oscillator

### 3.1. LC-Tank Schematic Design

The second architecture designed is based on an LC-tank resonator. This architecture bases its oscillation frequency on the filtering effect of an LC-tank, leaving for active components only the role of setting the feedback gain [25] and compensate for the loss of the inductor.

**Figure 5:**
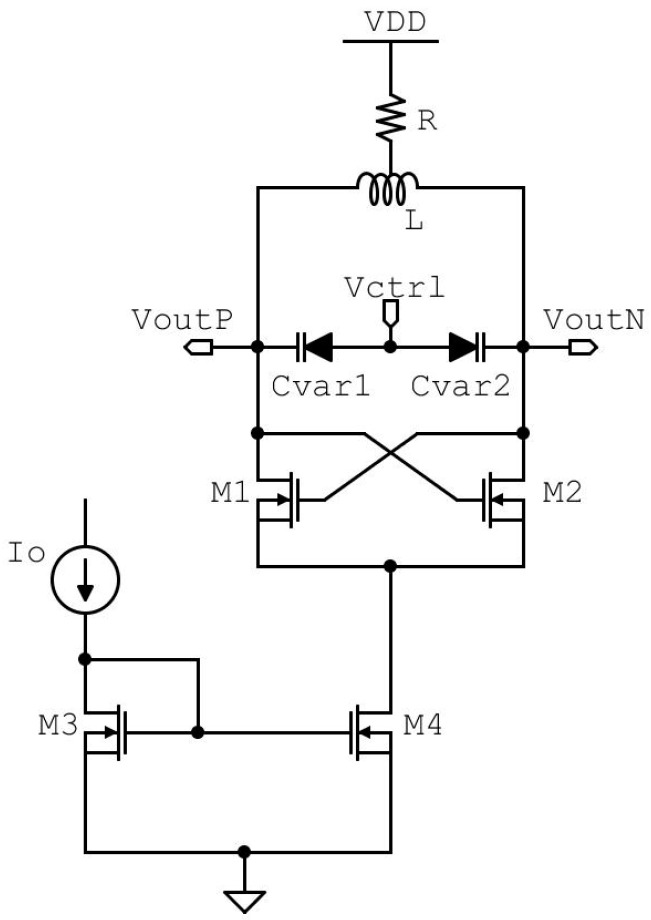
LC-tank VCO circuit schematic and a couple of varactors connected at the outputs.

The design guideline to respect Barkhausen oscillation criterion must be

(4) $$g_{m}>1/R_{P}$$

where $g_{m}$ is the value of the transconductance of the n-MOSFETs devices inside the cross-coupled cell, and $R_{P}$ is the parasitic resistance of the inductor [26]. Figure 5 shows the schematic of the LC-VCO designed to generate the target 6.25 GHz frequency. A polysilicon resistor is used to shift the output common-mode level at V_DD_/2, preventing the damaging or lifetime reduction of the low-voltage MOSFETs used for the cross-coupled pair.

This resistor is connected to the center tap of the symmetrical inductor chosen for its lower layout area than that of two separate inductors. In order to achieve the best frequency performance of this technology, the cross-coupled pair is sized using minimum length MOSFETs and a MOSFET width of 3.6 µm to guarantee a cell gain of at least 6 dB for start-up condition. The VCO bias current is controlled by the external current Io through the simple current mirror M3 and M4 with a current gain of 5, and the power consumption is less than 3 mW. The oscillation frequency of the LC-VCO is set by $f_{0}\;=\;1/\left(2\pi \sqrt{L(C+Cvar}\right))$ [26], where C is the equivalent capacitance due to the cross-coupled cell and the first stage of the output buffer, and Cvar is the capacitance of the accumulation-mode MOSFETs varactors connected at the controlled oscillator outputs. The Tuning Range (TR) is made with the control voltage Vctrl in the range from 0 V to V_DD_, and varactors assume, respectively, the value in the range from 629.6 fF to 197.6 fF, as shown in Figure 3b. A single varactor is composed of 120 fingers divided into 6 groups, and each finger is designed with 300 nm finger length and 1.2 µm finger width.

**Figure 6:**
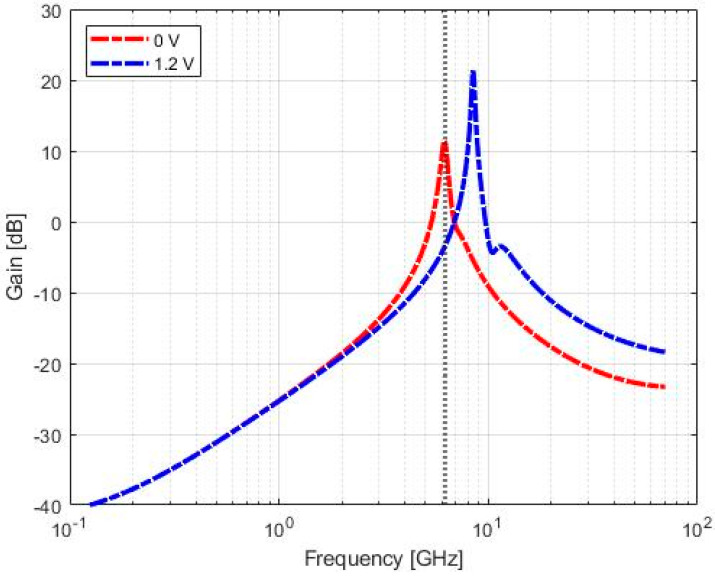
Frequency response simulated for minimum (**red line**) and maximum (**blue line**) values of the control voltage. The vertical marker indicates the target frequency of 6.25 GHz.

Figure 6 shows the simulated frequency response of the VCO for the two extreme values of the control voltage, and a minimum cell gain of about 10 dB for the minimum value of the control voltage, allowing to achieve a robust start-up condition for the oscillator.

### 3.2. LC-Tank Layout Design

**Figure 7:**
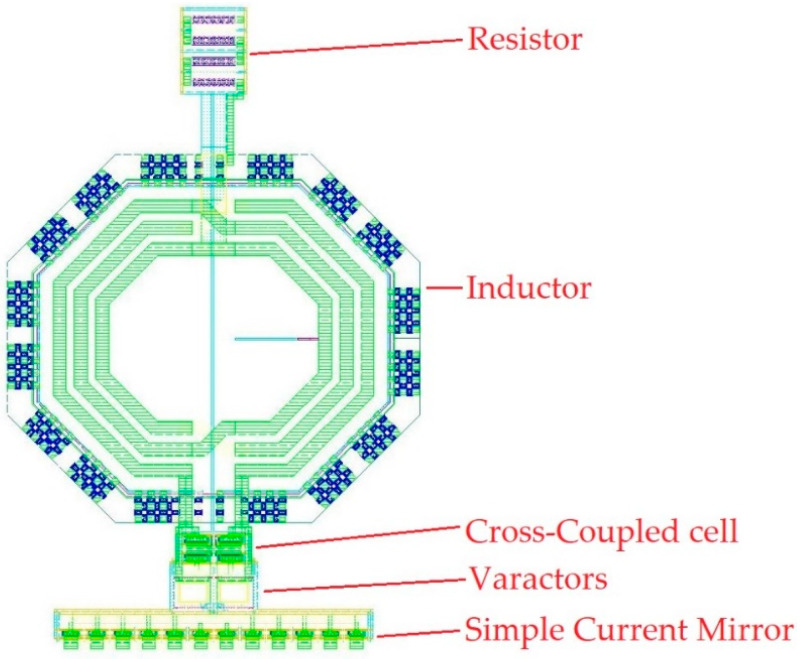
Full custom layout of the LC-tank VCO designed with Cadence Virtuoso [21].

The complete layout of the LC-VCO is shown in Figure 7, and it is composed of the simple current tail mirror, varactors, cross-coupled cell, inductor, and poly-silicon resistance from bottom to the top.

The current mirror is designed as a single strip, and a common centroid technique is adopted for the cross-coupled cell. Moreover, the minimum space allowed by technology rules is used, helping to increase matching. About 85% of the total area is occupied by the differential inductor (177 × 198 µm^2^) that has a quality factor of 20. It has been chosen with an odd number of turns because the two output terminals are on the same side of the cell, thus making the routing shorter with MOSFETs devices. Moreover, the single resistor connected to the center tap helps to reduce the metal connection length between the inductor and the cross-coupled cell.

The oscillator is designed to work properly in the temperature range −55 °C, +125 °C with 10% variations of current bias and voltage supply. The total layout area of the proposed LC-VCO is 308 × 198 µm^2^.

## 4. Simulations Results

### 4.1. Design Simulations

**Figure 8:**
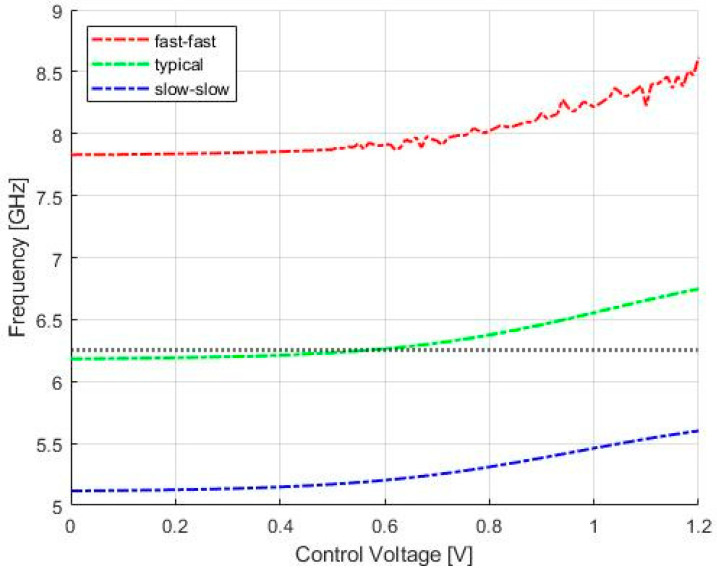
RO-VCO post-layout simulation of the free-running frequency versus control voltage in fast-fast (**red line**), typical (**green line**), and slow-slow (**blue line**) technology corner cases. The horizontal marker indicates the target frequency.

The small length size n-MOSFETs allowed to achieve high-frequency performance, but on the other hand, this choice increased the deviation of the device parameters from the typical condition. Although the frequency tuning was made with the use of accumulation-mode varactors, the frequency shift due to the technology simulations was so high that it could not be compensated using the control voltage. Figure 8 shows a post-layout simulation of the free-running oscillation frequency of the RO-VCO for the only three corners process. The frequency values were plotted versus an increasing value of the control voltage from the minimum to the maximum values. The oscillation frequency in the slow-slow corner case did not reach the 6.25 GHz frequency value required by the SpaceFibre standard, even using the maximum value of the control voltage. In the fast-fast corner case, the frequency was higher than the targeted frequency, even with the minimum value of the control voltage. Although the RO-VCO resulted as strongly dependent on the device parameters, in space applications, the best components should be selected.

**Figure 9:**
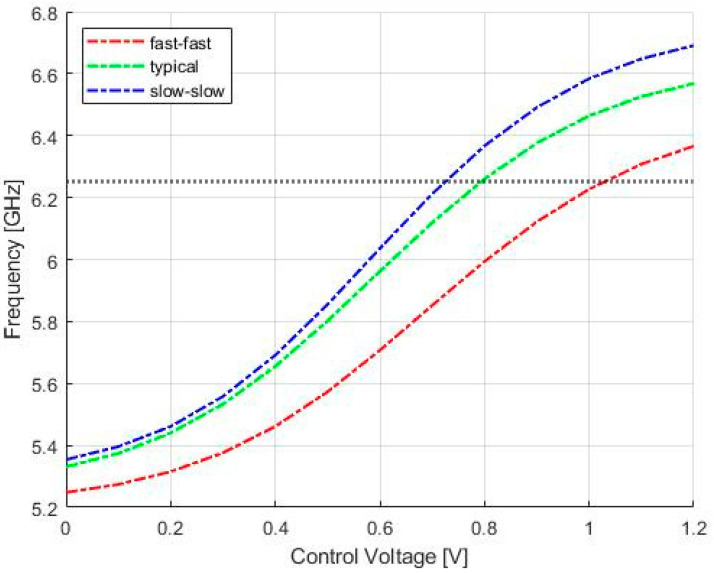
LC-tank VCO post-layout simulation of the free-running frequency versus control voltage in fast-fast (**red line**), typical (**green line**), and slow-slow (**blue line**) technology corner cases. The horizontal marker indicates the target frequency.

Although n-MOSFETs devices in the cross-coupled cell were designed with the minimum MOSFET length, the frequency shift in the LC-VCO, due to the technology simulations, could be recovered with the use of varactors and the control voltage. This can be seen by the curves in Figure 9, showing LC-tank VCO post-layout simulation of the free running-frequency versus control voltage in fast-fast (red line), typical (green line), and slow-slow (blue line) technology corner cases.

In addition to technology simulations, thus increasing the simulation realism, PVT (Process-Voltage-Temperature) simulations were performed by also changing temperature and supply voltage for both architectures. The SpaceFibre standard required the system to properly work under harsh conditions. In Table 1, the process and fabrication results are listed, respectively, in the third and fourth columns. The frequency variations were calculated as a variation from the nominal condition for temperature, supply voltage, and polarization current in each technology corner. The variations were obtained for temperature variations in the range −55 °C, 125 °C, and for ±10% supply voltage and polarization current deviations. Fabrication results were expressed as the frequency standard deviation σ, and data were obtained from the Monte Carlo simulations. Monte Carlo simulations were performed with 200 simulations in the nominal condition for each corner case considering process and mismatch variations.

**Figure 10:**
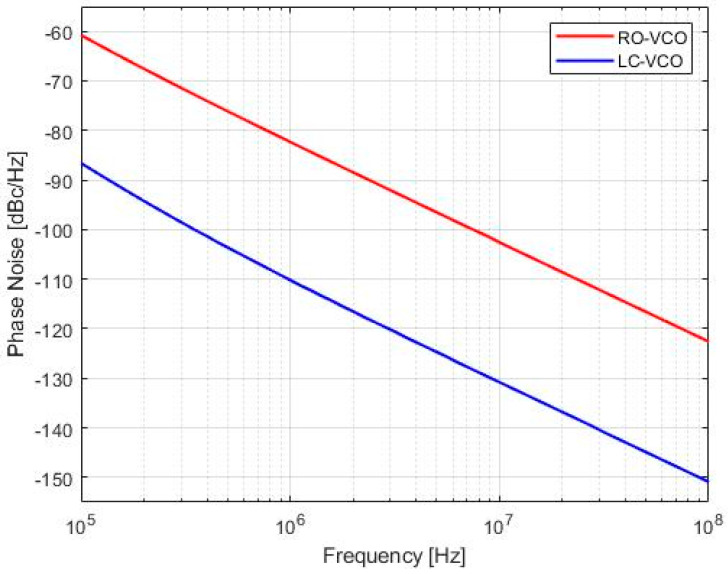
Phase noise simulated for RO-VCO (**red line**) and LC-VCO (**blue line**) at post-layout in typical conditions at the same working frequency of 6.25 GHz. These simulations were performed using the Cadence environment.

Figure 10 shows the simulated phase noise of the two architectures with the harmonic balance simulation in post-layout. Both VCOs were simulated at the same frequency, and the LC-VCO exhibited a better phase noise of about 30 dB than the other architecture (at 1 MHz offset, in Figure 9, there was a phase noise of −110 dBc/Hz for the LC-VCO vs. the −82 dBc/Hz for the RO-VCO). Device noise was considered in every simulation for both oscillator architecture and for all simulations performed in this work.

The integrated RMS jitter was calculated from Figure 10 in the bandwidth from 100 kHz to 10 MHz. The RMS jitter obtained was 9.51 ps and 0.44 ps for RO-VCO and LC-VCO, respectively. Moreover, the RO was more sensitive to temperature variations than the LC-VCO. The time-domain VCO stability was made with the use of the Allan variance [27,28], or two-sample variance, defined as

(5) $$\sigma_{x}^{2}\left(\tau \right)\;=\;\;\frac{1}{2}E\left[{\left(\bar{x_{2}}-\bar{x_{1}}\right)}^{2}\right]$$

**Figure 11:**
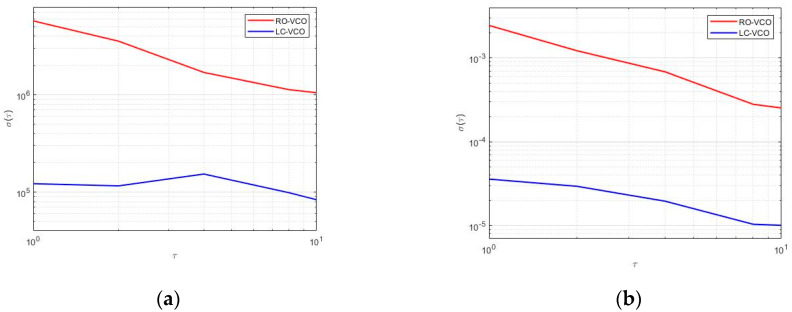
Allan deviation of (**a**) frequency and (**b**) amplitude for RO-VCO (**red line**) and LC-VCO (**blue line**) calculated in typical condition at the same oscillation frequency.

Figure 11 shows the Allan deviation, or *σ*-*τ* plot, calculated as the square root of Equation (5) when the VCO was in the steady-state oscillation. In particular, Figure 11a,b show the comparison between the Allan deviation in frequency and in amplitude, respectively, for both architectures. LC-VCO exhibited lower variations in frequency and amplitude than the RO-VCO.

### 4.2. Single Event Effect Simulations

The model used for SEE simulations and widely accepted by the scientific community [29,30,31] is shown in Equation (6), where *tinj* is the injection instant, *ta* is the collection time constant of the junction, *tb* is the ion track establishment time constant, and *Q* is the critical charge.

(6) $$ISET\;=\;\frac{Q}{ta-tb}\left[e^{-(\frac{t-tinj}{ta})}-e^{-(\frac{t-tinj}{tb})}\right]$$

**Figure 12:**
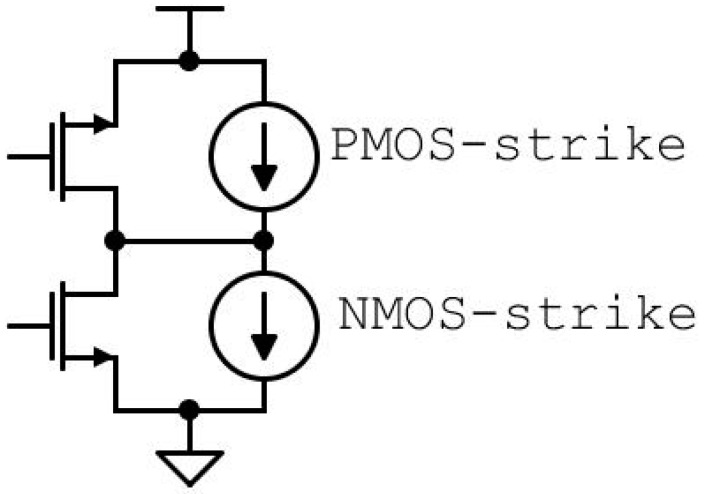
The model used for the correct stimulation of the P-N junction with the double exponential current pulses generators.

SEEs were modeled as double exponential current pulses at sensitivity nodes, and two different sets of values, with a Linear Energy Transfer (LET) of 5 and 60 MeV×cm^2^/mg, were used [32]. The two sets of values were expressed for different time constants versus critical charge Q and LET. The strike of an ionizing particle could be modeled by inserting a current pulse on each P-N junction, with the direction of the injected current depending on the device type [33], as shown in Figure 12. Moreover, the effects generated by the injected currents were strongly sensitive to the circuit conditions, requiring the analysis of the system in different states.

Both VCOs based their frequency tuning on accumulation-mode MOSFETs varactors, and the output nodes resulted as the most sensitive nodes in the whole architectures. Indeed, the strike of an ionizing particle generated a voltage variation in the node that was then translated in a frequency deviation by varactors. In this subsection, SEE simulations results are discussed, respectively, for RO and LC controlled oscillators.

**Figure 13:**
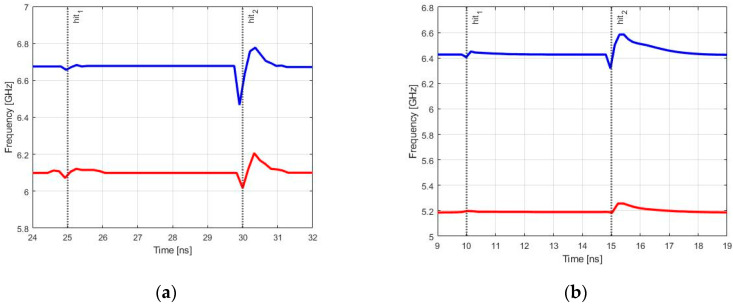
Two sets of values with a Linear Energy Transfer (LET) of 5 and 60 MeV×cm^2^/mg were used for Single Event Effect (SEE) simulations, and the free-running frequency was plotted for the control voltage value equal to 0 V (**red line**) and V_DD_ (**blue line**). (**a**) shows the free-running frequency in the RO-VCO, while (**b**) shows the free-running frequency in the LC-VCO.

**Figure 14:**
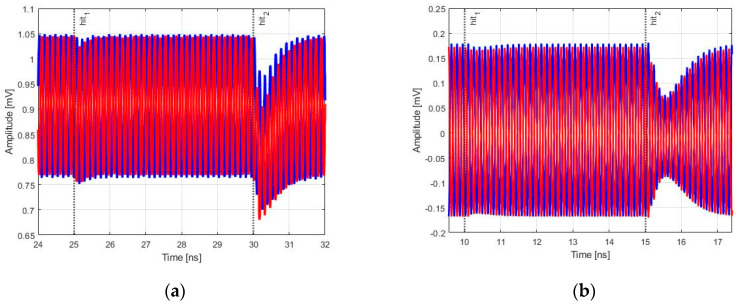
Differential amplitude variations due to the hit of the two LET values provided for the model. (**a**) shows the differential amplitude in the RO-VCO, while (**b**) shows the differential amplitude in the LC-VCO.

Figure 13 and Figure 14 show the effects generated by a particle strike for the two values of LET provided for the model in Equation (5). Particles with 5 and 60 MeV×cm^2^/mg are representant in the following figures with the label hit_1_ and hit_2_, respectively. The two exponential current generators excited sensitive nodes of RO-VCO at 25 ns and 30 ns, and the LC-VCO ones at 10 ns and 15 ns.

In Figure 13, the free-running frequency versus time is plotted for minimum (red line) and maximum (blue line) values of control voltage, and in Figure 14, the differential output amplitudes for the maximum value of the control voltage, respectively, for RO-VCO and LC-VCO are shown.

In Table 2, the data extracted from Figure 13 and Figure 14 are listed, where the column called clock cycles shows the number of clock cycles in which the frequency assumes different values respect to the nominal due to the strike of the particle. In the last two columns, the maximum variations for frequency and amplitude are reported.

As it is shown in the last two columns of Table 2, when the VCOs were hit by the ionizing particle with a LET of 5 MeV×cm^2^/mg (called hit_1_ in Table 2), both architectures showed nearly the same amplitude and frequency variations. Instead, when a particle with higher LET (called hit_2_ in Table 2) did hit the two VCOs, the amplitude variations of the LC-tank were greater than that of the RO, while the frequency variations were lower in the LC-tank-based architecture. This was despite that the LC architecture used one order greater varactor capacitances than RO one. This greater frequency deviation in RO-VCO was due to the frequency relationship with capacitance, which was 1/C for the RO-VCO and $1/\sqrt{C}$ for the LC-VCO. This attested that a small capacitance variation generated a huge frequency variation in the RO-VCO, as highlighted in Table 2.

n-MOSFETs devices in both architectures were designed with the minimum channel length targeting high-frequency applications, but a maximum number of fingers and an oversized MOSFET width were used to increase the parasitic capacitance of devices. Although this SEE mitigation technique increased the silicon area and reduced the tuning range, it increased the SEE tolerant property of both VCOs. Indeed, following the simple rule V = Q/C, if the capacitance value was increased for a fixed value of charge, then a lower variation of the voltage occurred. Moreover, guard rings and deep n-wells were adopted to isolate the devices by the charge generated in the substrate during a particle strike. Indeed, if an ionizing particle passed through the device, electron-hole pairs could be generated, which, thank to guard rings and deep n-wells, were rapidly collected, avoiding the activation of parasitic latch-up.

## 5. Comparison vs. the State-of-the-Art

A state-of-the-art comparison of voltage controlled oscillator designed in 65 nm technology is made in Table 3. In works [9,10], the PLLs were based on a RO and LC-VCO. They were irradiated up to 300 krad TID level compliant with SpaceFibre protocol and tested with different protons. Their working frequency did not meet that required by the SpaceFibre standard, and the aim of this work was to investigate the behavior of these two well-known architectures at a higher frequency. In [34], a VCO based on LC tank was optimized against SEEs, and in [35], a three stages ring oscillator was designed targeting Bluetooth front-end applications, but no process simulations were performed. Another solution presented in [36] was based on a Colpitts architecture for mm-wave applications.

## 6. Conclusions

In this work, the comparison between two VCO architectures designed in a commercial 65 nm technology was made. Targeting high-frequency space applications, as the SpaceFibre protocol, a CML approach was adopted for the design of the RO-VCO. CML architecture was preferred, targeting high frequency, thanks to its lower voltage swing than a CMOS. The RO-VCO was an appetible VCO configuration in terms of technology scaling, high-integration density, and area occupancy, which was about 35% of the total silicon area required for the LC-VCO. Although the RO-VCO resulted as strongly dependent on the device parameters, in space applications, the best components should be selected. To overcome the effects of the device parameters deviation on the oscillation frequency, an LC-tank VCO architecture was designed. This architecture, despite its large area, mainly occupied by the inductor, presented promising performances in terms of the frequency range, covering the 5.35 GHz to 6.55 GHz range, in the typical case, with a control voltage swing of V_DD_. SEE simulation results highlighted the output nodes as the most sensitive nodes for both VCOs, for the effects due to the varactors. Although the LC-tank VCO used one order greater varactor values than RO, and the ionizing particle hits generated higher amplitude variation on its output signals, the frequency variations of this VCO were lower than that showed by the RO architecture, thanks to the different relationship between frequency and capacitance. In the literature, VCOs based on Colpitts architecture for space applications are not available because of their large silicon area. The LC system, whose layout is shown in Figure 7, would be integrated into a 1 mm^2^ chip containing a SERDES (Serializer-Deserializer) to test system-level performance. The whole chip would be electrically tested in standard condition, then it would be exposed to X-rays to achieve the 300 krad TID and to heavy ions for SEE characterization.

## Figures and Tables

**Table 1:** Frequency variations and standard frequency deviation, respectively, for PVT (Process-Voltage-Temperature) and Monte Carlo simulations.

Oscillator	Technology Corners	Frequency Variations	Standard Deviation σ (Hz)
RO	slow-slow	31.46%	0.63
	typical	28.34%	0.89
	fast-fast	15.72%	3.34
LC	slow-slow	9.01%	99.69
	typical	9.07%	578.5
	fast-fast	7.04%	132.2

**Table 2:** Variations due to a SEE for RO-VCO and LC-VCO.

Oscillator	Hit	Control Voltage	Clock Cycles	Frequency Variations	Amplitude Variations
RO	hit_1_	0	6	0.61%	−1.65%
		V_DD_	3	0.27%	−2.93%
	hit_2_	0	9	1.53%	−15.38%
		V_DD_	9	3.12%	−16.27%
LC	hit_1_	0	6	0.11%	1.55%
		V_DD_	13	0.46%	3.17%
	hit_2_	0	15	1.24%	43.80%
		V_DD_	24	2.45%	57.49%

**Table 3:** State-of-the-art comparison.

Ref.	Frequency (GHz)	Power Consumption (mW)	SEE Tolerant	Architecture	Area (mm^2^)
[9]	0.2–1.2	n.a.	tested	Ring	n.a.
[10]	2.2–3.2	6	tested	Ring and LC	n.a.
[34]	2.25–2.88	1.8	tested	LC tank	n.a.
[35]	3.7–6.5	2	no	Ring	0.011
[36]	23.8–29.1	2.3	no	Colpitts	0.221
**This work**	6.20–6.75	18	simulated	Ring	0.021
**This work**	5.35–6.55	<3	simulated	LC tank	0.061
